# Epinephrine and the paradox of Kounis syndrome: a case series and review of management

**DOI:** 10.1093/ehjcr/ytaf414

**Published:** 2025-09-03

**Authors:** Mohammad Hamideh, Alec Rhodes, Aswin Srinivasan, Shudipan Chakraborty, Christopher Hawkins, Aakash Kumar, Hafez Golzarian

**Affiliations:** Internal Medicine Residency Program, HCA Houston Healthcare—Kingwood/University of Houston College of Medicine, 22999 US-59 N, Houston, TX 77339, USA; Department of Cardiovascular Disease, HCA Houston Healthcare—Kingwood/University of Houston College of Medicine, 1313 Hermann Dr, Houston, TX 77004, USA; Department of Cardiovascular Disease, HCA Houston Healthcare—Kingwood/University of Houston College of Medicine, 1313 Hermann Dr, Houston, TX 77004, USA; Cardiovascular Medicine Fellowship—University of Nevada, 4505 S. Maryland Pkwy, Las Vegas, NV, USA; Department of Internal Medicine, Mercy Health—St. Rita’s Medical Center, 730 West Market Street, Lima, OH 45801, USA; Department of Internal Medicine, Mercy Health—St. Rita’s Medical Center, 730 West Market Street, Lima, OH 45801, USA; Department of Cardiovascular Disease, HCA Houston Healthcare—Kingwood/University of Houston College of Medicine, 1313 Hermann Dr, Houston, TX 77004, USA

**Keywords:** Anaphylaxis, Kounis syndrome, Mechanical circulatory support, Cardiogenic shock, Haemodynamics, Case series

## Abstract

**Background:**

Epinephrine, the primary treatment for anaphylaxis, may paradoxically worsen underlying myocardial ischaemia due to its vasoconstrictive effects and potential to trigger vasospasm during an inflammatory response.

**Case Summary:**

We present two cases of Kounis syndrome—one in a patient who developed Prinzmetal angina after exposure to shellfish and another who experienced cardiac arrest after receiving intramuscular epinephrine for an anaphylactic reaction to a wasp sting.

**Discussion:**

These cases emphasize the potential for anaphylaxis to precipitate acute coronary syndrome which can paradoxically be exacerbated by the routine administration of epinephrine in susceptible individuals, underscoring the importance of heightened vigilance and consideration of alternative therapies when Kounis syndrome is suspected.

Learning points

- The successful treatment of Kounis syndrome necessitates a multimodal approach that carefully balances the management of both the allergic and cardiovascular components of this complex condition.
- Epinephrine, while the first-line treatment for anaphylaxis, can paradoxically worsen coronary supply in susceptible individuals, potentially exacerbating the clinical course in patients who concomitantly develop acute coronary syndrome.

## Introduction

Kounis syndrome is a condition where acute coronary events occur due to inflammatory mast cell activation triggered by an allergen, such as in hypersensitivity reactions and anaphylaxis. The inflammatory mediators released can precipitate coronary artery spasm or contribute to plaque erosion and rupture during allergic reactions. Epidemiological evidence suggests that Kounis syndrome affects approximately 1.1% of patients hospitalized for allergic, hypersensitive, or anaphylactic episodes in the USA, with a mortality rate of around 7.0%.^1^ It can occur in individuals with or without underlying coronary artery disease. Type 1 Kounis syndrome, typically involving coronary vasospasm, is more commonly seen in patients with normal or near-normal coronary arteries, whereas Type 2 occurs in those with pre-existing coronary artery disease. Although traditional cardiovascular risk factors such as hyperlipidaemia, diabetes, hypertension, and smoking may be present, they are not causative. Due to frequent underdiagnosis, the true prevalence and association with conventional risk factors remain unclear; however, Kounis syndrome should be considered across a broad clinical spectrum regardless of cardiovascular history. While epinephrine is the recommended first-line treatment for anaphylaxis, we present two cases that highlight the potential for epinephrine to play a role in provoking acute coronary syndrome (ACS) during an allergic reaction by means of its vasoconstrictive properties in select patients. This adverse effect is often underreported, emphasizing the need for heightened awareness and consideration of alternative therapies when Kounis syndrome is suspected.

## Summary figure

**Figure:**
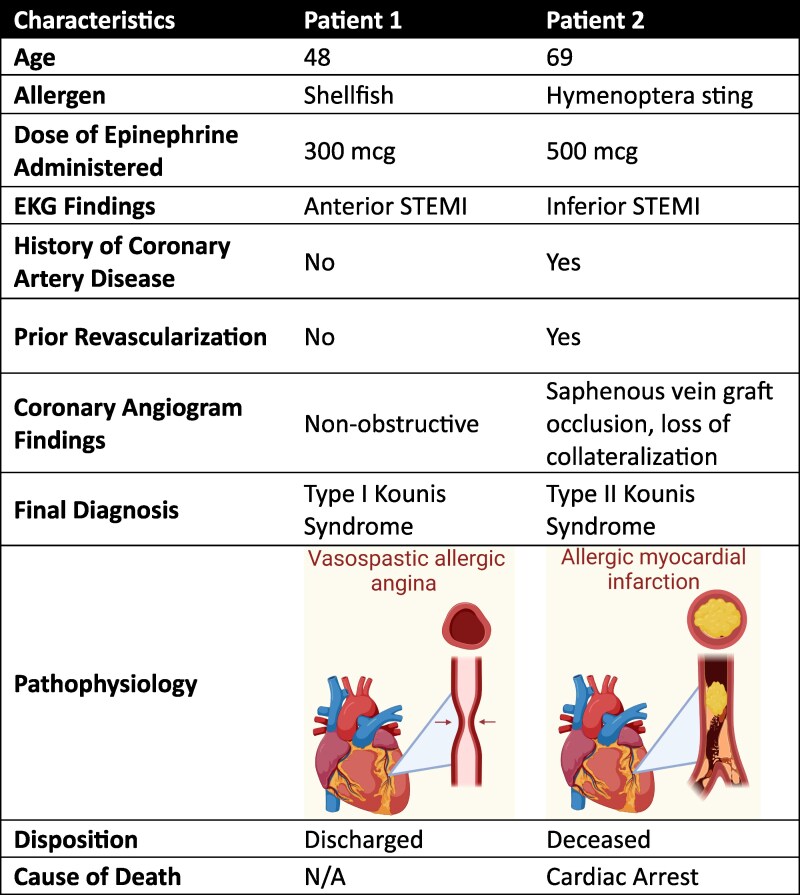


## History of presenting illness

### Patient 1

**Figure 1:**
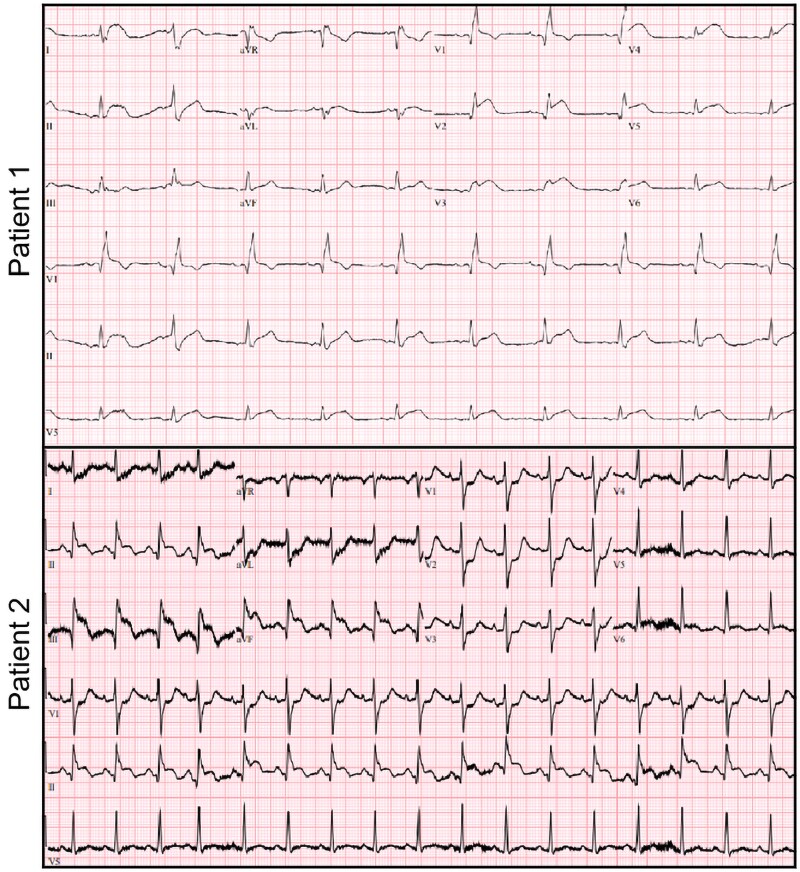
Electrocardiograms on arrival. Top panel demonstrates the ST segment elevations noted in anterior leads for Patient 1. The bottom panel reveals ST elevations in the inferior leads with reciprocal changes in anterior leads for Patient 2.

**Figure 2:**
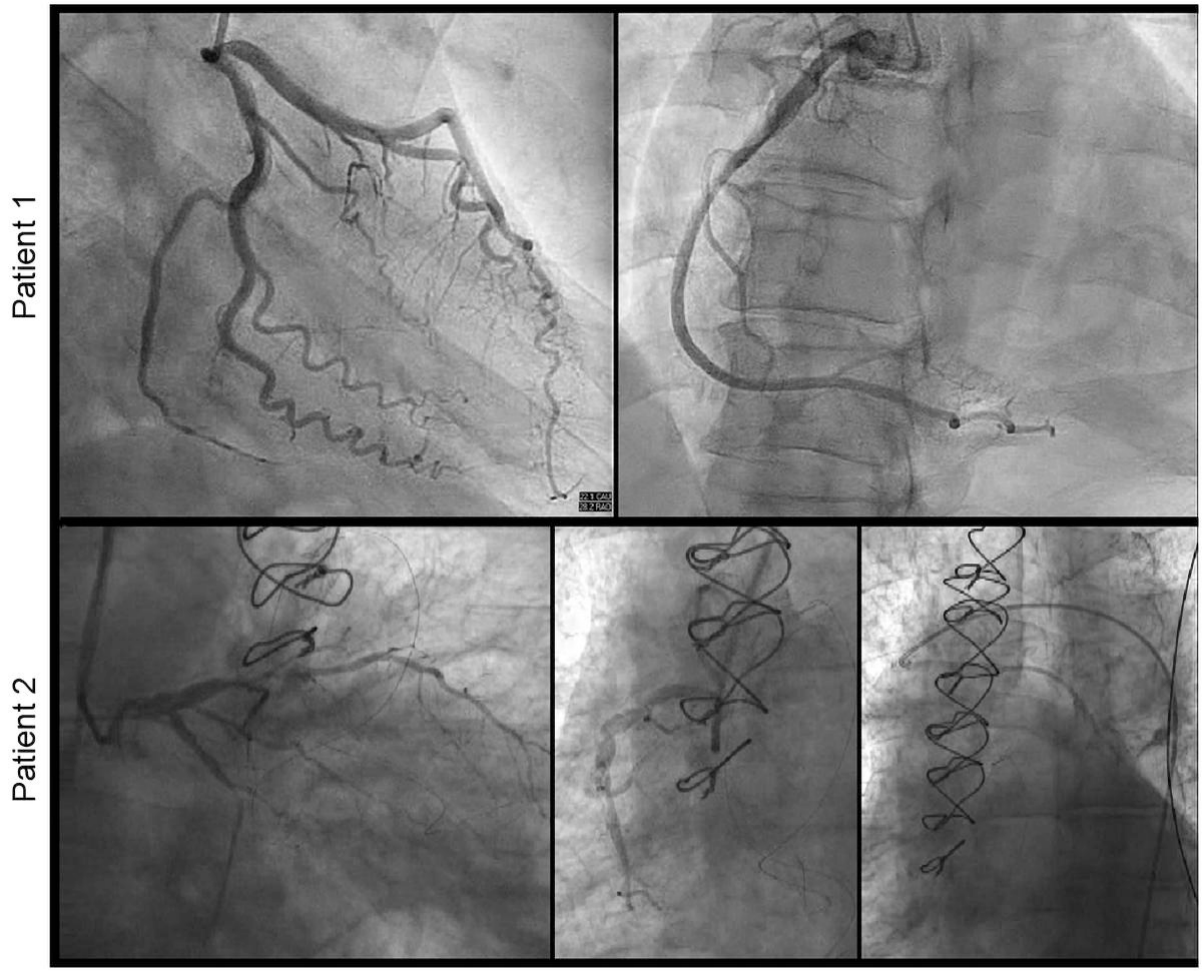
Left heart catheterizations. Top panels demonstrating the tortuous but widely patent coronaries in right anterior oblique views for Patient 1. Bottom panel revealing diffuse multivessel coronary disease with complete occlusion of the saphenous vein graft and no collateralization.

A 48-year-old female with history of hypertension, Class II obesity, shellfish allergy, and prediabetes presented to the emergency department with complaints of new-onset chest tightness, radiating to her left shoulder and jaw within minutes after prophylactic self-administration of 0.3 mg of intramuscular epinephrine at a restaurant where she was exposed to shellfish. Chest pain was waxing and waning in nature and would last several seconds. A 0.4 mg of sublingual nitrogen aborted the symptoms. Vital signs and physical exam were unremarkable. During triage, ST elevations were noted on her electrocardiogram (*Figure 1*). Initial serum troponin levels were elevated at 2932 pg/mL (reference: ≤15 pg/mL). We administered 324 mg aspirin, 0.4 mg sublingual nitroglycerin, and continuous unfractionated heparin, before promptly taking her to the catheterization lab. Chest pain terminated with sublingual nitroglycerin. To our surprise, during coronary angiography, all coronaries were found to be patent (*Figure 2*) consistent with the diagnosis of myocardial infarction with nonobstructive coronary arteries. Her troponin levels peaked at 8349 pg/mL and then began to trend down. She was monitored overnight and underwent echocardiogram which was unremarkable (see Supplementary material online, *Video S1*) as well. Given the history of presentation and workup, Type 1 Kounis syndrome was suspected. The remainder of her course was uneventful, and she was subsequently discharged home in stable medical condition.

### Patient 2

A 69-year-old male with chronic obstructive pulmonary disease, hymenoptera venom allergy, coronary artery disease status post prior coronary artery bypass grafting, hypertension, and hyperlipidaemia was brought to the emergency department after experiencing cardiac arrest following the administration of intramuscular epinephrine for an anaphylactic reaction to a wasp sting while working in his yard. His wife reportedly had called emergency services. Upon arrival of first responders, he was lethargic and hypotensive, with a systolic blood pressure of 84 mmHg. Emergency personnel administered 0.5 mg intramuscular epinephrine, and his blood pressure transiently improved to 170 mmHg shortly afterwards. However, the patient then began complaining of chest tightness and became profusely diaphoretic. En route to the hospital, he rapidly deteriorated into a state of hypoxic respiratory failure and cardiogenic shock, with subsequent cardiac arrest. Spontaneous circulation was restored after 20 min of cardiopulmonary resuscitation in the emergency department. A 12-lead electrocardiogram showed an acute inferolateral ST elevation myocardial infarction (*Figure 1*). The patient was promptly transferred to the cardiac catheterization laboratory, where left heart catheterization revealed complete occlusion of his saphenous vein graft (*Figure 2*, Supplementary material online, *Video S2*). The graft had completely shut down and was not amenable to percutaneous interventions despite numerous attempts. Additionally, there was concern for collapse of collateral vessels. Two-dimensional echocardiography revealed left atrial dilation, hypokinesis of the basal and inferolateral wall, and an estimated ejection fraction of 15–20% (see Supplementary material online, *Video S3*). He was placed on venoarterial extracorporeal membrane oxygenation to provide mechanical circulatory support and received inotropes and multiple pressors to maintain adequate perfusion. Thereafter, he received comprehensive supportive care in the intensive care unit which included invasive haemodynamic monitoring and continuous renal replacement therapy. Unfortunately, in the days that followed, he developed severe liver failure and disseminated intravascular coagulation. Once anoxic brain injury was confirmed, the family elected to withdraw care.

## Discussion

Epinephrine is the primary treatment for anaphylaxis due to its ability to relax airway muscles, cause vasoconstriction to maintain blood pressure, stimulate increased heart rate through beta-adrenergic effects, and prevent the release of histamine, which can further worsen the allergic reaction. At low to moderate doses, epinephrine primarily works on G-protein–coupled β2-adrenergic receptors, causing vasodilation through mediation by Class C L-type calcium channels. However, at higher doses, it acts on α1-receptors, resulting in systemic and coronary vasoconstriction/vasospasm, particularly in predisposed individuals.

**Figure 3:**
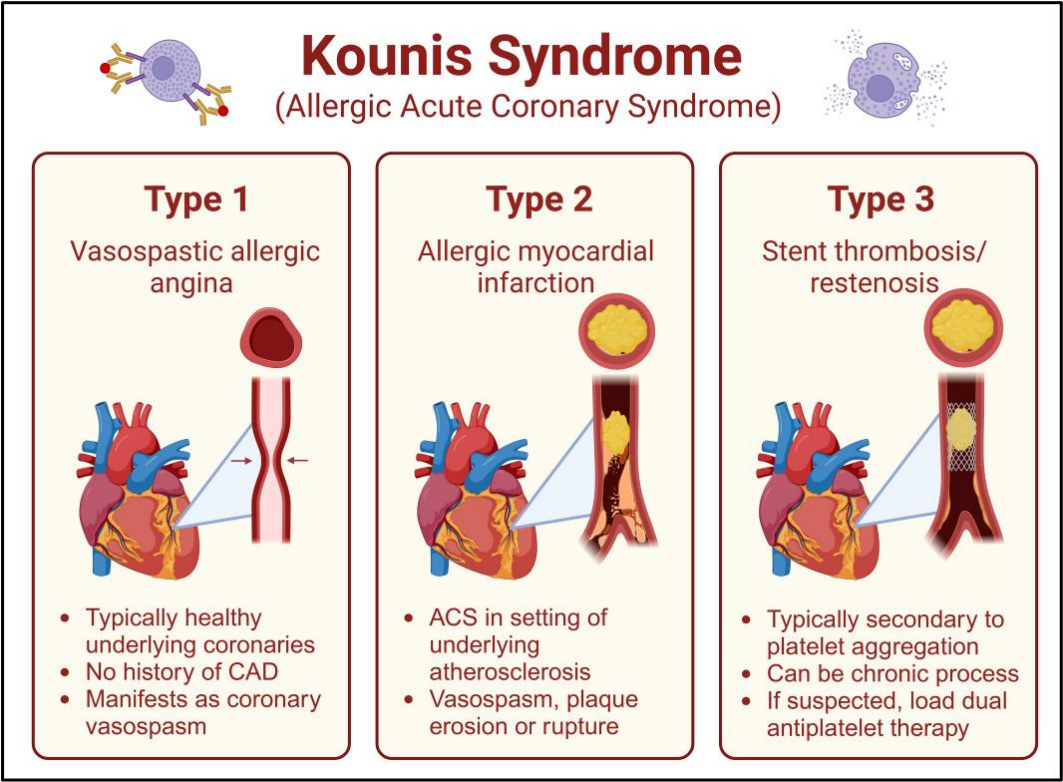
The variants of Kounis syndrome. Illustration created with BioRender.com.

Kounis syndrome was first identified in 1991 as concurrence of ACS with allergic or anaphylactic reactions, primarily due to the release of inflammatory mediators from activated mast cells. These inflammatory mediators include a wide variety of interleukins, arachidonic acid by-products, histamine, and platelet activating factors. The syndrome has since been classified into three distinct variants: Type 1, characterized by acute coronary events resulting from coronary spasm; Type 2, characterized by coronary thrombosis; and Type 3, characterized by in-stent thrombosis or restenosis (*Figure 3*).

Leukotrienes have been well-established to induce coronary artery disease and syndromes.^2^ Similarly, tryptase, an inflammatory molecule released during hypersensitivity reactions, also has potential to propagate plaque erosion and rupture.^3,4^ During such heightened inflammatory responses, such disastrous thrombotic events should be anticipated. Our cases highlight the diagnostic and therapeutic challenges associated with this disease process given the complex interplay between allergic and cardiovascular systems. It was evident that both patients experienced anaphylactic reactions. While epinephrine is the cornerstone of treatment, it should be known that its vasoconstrictive properties can paradoxically exacerbate coronary vasospasm, potentially inducing ACS in susceptible individuals.^5^ The cases underscore the delicate balance required when administering epinephrine in patients with suspected Kounis syndrome. The development of angina or ACS in the setting of anaphylaxis or epinephrine administration involves overlapping risk factors that may make it very difficult to distinguish. Below, we have tabulated risk factors that favour each type of clinical presentation (*Table 1*).

**Table 1:** Risk factors favouring Kounis syndrome or epinephrine-induced acute coronary syndrome

Risk factors	Favours isolated anaphylaxis-induced angina or ACS	Favours superimposed epinephrine-induced ACS
Age >65	✅	✅
BMI ≤ 20	❌	✅
Pre-existing coronary artery disease	✅	✅
Prior revascularization	✅	✅
Active hypertension	❌	✅
Prinzmetal (variant) angina	✅	✅
Diabetes mellitus	✅	✅
Tobacco smoking	✅	❌
High-dose epinephrine (≥500 mcg)	❌	✅
Onset of cardiac symptoms prior to epinephrine administration	✅	❌
Stimulants	❌	✅
Alpha-agonists	❌	✅
Beta-blockers	✅	✅
Levothyroxine	❌	✅
Tricyclic antidepressants	❌	✅

Abbreviations: ACS, acute coronary syndrome; BMI, body mass index; mcg, micrograms.

Although nitrates and alternative vasodilators have been shown to help in Kounis syndrome (especially Type 1), these may worsen the state of shock, and the immediate priority is often reversing the life-threatening anaphylaxis first. A multimodal approach with antihistamines, anti-inflammatory agents, and corticosteroids has been proposed in the literature, though unfortunately the evidence is limited.^6^ The potential for first responders to identify patients at high risk for Kounis syndrome should be further explored. One proposed strategy involves the empirical administration of a combination of treatments in a cocktail consisting of high-dose aspirin, antihistamines, non-steroidal anti-inflammatory drugs, and thrombin inhibitor, alongside the standard intramuscular epinephrine injection upon their initial arrival to the scene. We do believe patients with prior percutaneous interventions should especially be empirically loaded with dual antiplatelet therapy.^5–7^ However, data regarding these other agents remains anecdotal, even controversial, and cannot be generalizable yet.

Even the routine administration of aspirin remains a subject of debate among some practitioners, particularly given its potential to induce mast cell activation in susceptible individuals. For instance, aspirin intolerance, encompassing conditions like aspirin-exacerbated respiratory disease, affects a notable proportion of asthmatics (up to 7%) and individuals with chronic urticaria (20–30%).^8^ Nevertheless, the clinical consensus leans towards its overall benefit, with its advantages outweighing the potential risks in managing this condition, particularly as it has demonstrated efficacy in reducing prostaglandin D2 levels during mastocytosis.^9^

Heparin's role in Kounis syndrome is also multifaceted, as it can paradoxically act as both a trigger and a treatment. While routine heparin administration in ACS is standard practice in many institutions due to its perceived benefits, it is not without risks. Heparin-induced thrombocytopenia, a serious complication, has been linked to Kounis syndrome, potentially exacerbating the anaphylactic cascade.^10^ HIT can lead to platelet and monocyte activation, subsequently causing thrombin formation, particularly concerning in Type 2 and 3 Kounis syndrome variants. This paradox can result in both bleeding complications and unexpected in-stent thrombosis. Furthermore, certain venoms, like those of wasps, possess anticoagulant properties that lead to ‘heparinization’ and the prolonging activated partial thromboplastin times, a phenomenon well-documented in medical literature.^11–13^ Therefore, cautious anticoagulation is advised for select patients, and alternative thrombin inhibitors like bivalirudin or argatroban may ultimately be preferable to heparin in these cases.

Ultimately, the management of anaphylaxis in patients with elevated cardiovascular risk necessitates a tailored approach, integrating standard protocols with heightened vigilance due to potential complications such as coronary vasospasm and myocardial infarction. *Table 2* reveals our proposed algorithm, incorporating guidelines from the World Allergy Organization, European Resuscitation Council, American Heart Association, as well as the few existing reports of epinephrine-induced ACS in the literature.

**Table 2:** Proposed management in Kounis syndrome and high-risk patients

**Step 1**	**Recognition** Look for: Skin: hives, flushing Respiratory: wheezing, bronchospasm, stridor, dyspnoea Cardiovascular: hypotension, dizziness GI: vomiting, abdominal pain
**Step 2**	**Remove allergen and call emergency services immediately**
**Step 3**	**Epinephrine administration** Administer i.m. epinephrine into the anterolateral thigh as soon as possible Repeat i.m. dose if symptoms persist after 5–15 min.
**Step 4**	**Proceed to the nearest emergency department** (centres with advanced cardiac services preferred) **Identify risk factors and cardiovascular risk-averse patients (*Table 1*)** If risk factors for ACS or arrhythmogenesis or prior history of revascularization present: Monitor for signs and symptoms of ACS Obtain 12-lead ECG for all patients with cardiovascular disease. Prophylactically load with aspirin 324 mg for select patients (If recent revascularization or stent placement within 3 months, load with dual antiplatelet therapy) Attach external defibrillator and monitor rhythm and vital signs If blood pressure stable or elevated, consider intravenous nitroglycerin If pro-arrhythmogenic, consider prophylactic administration of i.v. amiodarone 150 mg bolus Administer glucagon if on beta-blockers and unresponsive to epinephrine **Additional supportive measures:** Start intravenous normal saline Consider intravenous bivalirudin or argatroban when anticoagulation desired Administer bronchodilators (albuterol) for bronchospasm Administer antihistamines and intravenous steroids Lay flat if hypotensive (left lateral recumbent for pregnant patients) Initiate high-flow O₂ for respiratory distress or ACS
**Step 5**	**Wallet medical ID card at discharge** Patient with Kounis syndrome or cardiovascular adverse effects from epinephrine should be provided with Wallet Medical ID Card on discharge indicating such

Abbreviations: ACS, acute coronary syndrome; ECG, electrocardiogram; GI, gastrointestinal; i.m., intramuscular; i.v., intravenous.

For Patient 2, his rapid deterioration despite aggressive interventions, including percutaneous coronary intervention, mechanical circulatory support, and respiratory support, highlights the severity of Kounis syndrome, particularly in its Type 2 and 3 variants. These variants, involving plaque rupture or erosion and coronary thrombosis, respectively, are associated with higher mortality rates, especially in patients with pre-existing coronary artery disease. The unsuccessful percutaneous intervention attempts likely reflect the complexity of the coronary anatomy in a post-bypass patient with a failing graft and loss of vulnerable and frail collaterals, complicated by the acute allergic reaction and inflammation cascade that rendered the patient’s unfortunate fate.

These cases reinforce the importance of prompt recognition of individuals susceptible to Kounis syndrome management by our first responders. Early diagnosis and aggressive management of both the allergic reaction and the ensuing ACS are crucial. While the outcome for one of the patients was unfavourable, the use of epinephrine in anaphylactic shock is lifesaving and should not be discouraged. We hope these cases contribute to the growing body of literature emphasizing the need for heightened awareness and refined treatment strategies for Kounis syndrome. Further research is needed to better understand the pathophysiology, optimize treatment protocols, and improve patient outcomes in this rare but potentially fatal condition. This includes exploring treatment optimization for anaphylaxis in high-risk patients and developing risk stratification tools to guide management decisions.

## Conclusion

Kounis syndrome is a rare yet serious condition where ACS arises secondary to allergic or hypersensitivity reactions. It is being increasingly recognized as a crucial differential diagnosis for patients presenting with ACS and a history of hypersensitivity. Ongoing efforts aiming to elucidate its pathophysiology, optimize treatment strategies, and investigate long-term prognosis will be instrumental in enhancing the management of affected individuals and raising awareness among healthcare professionals. Epinephrine is a lifesaving medication in patients with anaphylactic shock. However, physicians should be cognizant of this complication in patients with risk factors for Kounis syndrome. The successful treatment of Kounis syndrome necessitates education and prompt recognition of susceptible individuals by first responders and clinicians with a timely approach that carefully balances the management of both the allergic and cardiovascular components of this complex condition.

## Supplementary Material

ytaf414_Supplementary_Data

## Data Availability

The data involved in this case study are available from the authors upon request.

## Lead author biography

Dr. Mohammad N Hamideh is an internal medicine resident at HCA Houston Healthcare in Houston, Texas (USA). He has clinical interests in interventional cardiology, heart failure, and critical care.
